# Pain sensitivity in posttraumatic stress disorder and other anxiety disorders: a preliminary case control study

**DOI:** 10.1186/s12991-014-0031-1

**Published:** 2014-11-18

**Authors:** Sheeva Mostoufi, Kathryn M Godfrey, Sandra M Ahumada, Nazia Hossain, Titus Song, Lisa Johnson Wright, James B Lohr, Niloofar Afari

**Affiliations:** San Diego State University/University of California, San Diego Joint Doctoral Program in Clinical Psychology, 6363 Alvarado Court, Suite 103, San Diego, CA 92120-491 USA; VA Center of Excellence for Stress and Mental Health (CESAMH) and the VA San Diego Healthcare System, 3350 La Jolla Village Drive, San Diego, CA 92161 USA; Institute of Child Development, University of Minnesota, 51 East River Parkway, Minneapolis, MN 55455 USA; Department of Psychiatry, University of California, 9500 Gilman Drive, 0737, La Jolla, San Diego, CA 92093 USA; VA Northern California Healthcare System, 10535 Hospital Way, Mather, CA 95655 USA

**Keywords:** PTSD, Anxiety, Pain sensitivity, Chronic pain, Comorbidity

## Abstract

**Background:**

Despite substantial research on the comorbidity of anxiety disorders including posttraumatic stress disorder (PTSD) and chronic pain, little is known about the mechanisms underlying these conditions that might be potentially similar. Evoked pain sensitivity is one factor that has been associated with several pain conditions which might also have relevance to anxiety disorders and PTSD. The aim of this preliminary study was to examine evoked pain sensitivity in PTSD compared to other anxiety disorders and in control participants.

**Method:**

The study used a cross-sectional case-control design in which participants completed a battery of questionnaires and structured interview and underwent cold pressor testing.

**Results:**

Of 61 total participants, those in the PTSD (*n* =16) and other anxiety groups (*n* =12) endorsed significantly higher levels of psychological symptoms and poorer health functioning than control participants (*n* =33). The linear trend across baseline, threshold, and tolerance pain ratings from the cold pressor task significantly differed between participants with PTSD and the other anxiety and control groups suggesting lower pain sensitivity to a standardized stimulus of pain in individuals with PTSD.

**Conclusions:**

These findings are similar to some of the prior research and suggest that individuals with PTSD may exhibit lower cold pain sensitivity compared to those with other anxiety disorders. There is a need for future research to determine explanatory mechanisms.

## Background

A substantial body of literature has documented that anxiety disorders such as posttraumatic stress disorder (PTSD) and chronic pain often occur concurrently [1-5]. An estimated 11–60% of patients with chronic pain report the co-occurrence of various anxiety disorders [6]. Individuals with PTSD and chronic pain report higher ratings of psychiatric distress, greater disability, and more intense pain compared to those with PTSD or chronic pain only [7-9]. Despite substantial research on this comorbidity, little is known about the mechanisms underlying these conditions that might be potentially similar across anxiety disorders including PTSD and chronic pain. Evoked pain sensitivity is one factor that has been associated with several pain conditions and extensively examined in the pain literature [10,11], which may also have relevance to anxiety disorders and PTSD.

Evoked pain sensitivity is generally described in terms of *pain threshold*, which is the lowest stimulus intensity of pain that is detected by an individual, and *pain tolerance*, which is the maximum pain intensity one is willing to stand. Increased pain sensitivity is typically conceptualized along both perceptual (i.e., higher pain ratings) and behavioral (i.e., lower time to tolerance) domains, with both sets of measurements reflecting independent indicators yet complementary information on one’s sensitivity to evoked pain stimuli [12,13]. Cross-sectional studies of patients with fibromyalgia [11] and low back pain [10], for example, have found increased sensitivity to painful stimuli as measured in both perceptual and behavioral domains. Further, increased pain sensitivity has been linked to a higher incidence of temporomandibular pain disorders in a prospective cohort study [14]. Although the literature is largely cross-sectional, making it difficult to determine whether increased pain sensitivity precedes or follows chronic pain, these findings suggest that greater sensitivity to painful stimuli may play a role in the development and maintenance of chronic pain.

There is a small body of literature examining pain sensitivity in PTSD and other anxiety disorders. Interestingly, despite the substantial comorbidity of PTSD with chronic pain conditions, individuals with PTSD have been found to endorse “hyposensitivity” or lowered perception of pain in evoked pain paradigms [15-17]. However, the one study that compared patients with PTSD to other anxiety disorders had mixed results, perhaps because individuals with chronic pain were not excluded [18]. While individuals with PTSD (*n* =32) were found to rate painful stimuli as more intense than other anxiety (*n* =29) and control (*n* =20) groups suggesting increased pain sensitivity on the perceptual indicators, they exhibited higher thresholds and, therefore, lowered sensitivity on the behavioral indicators of pain [18]. Thus, there is some reason to believe that individuals with PTSD may have altered pain sensitivity compared to both healthy individuals and those with other anxiety disorders. However, given the known association of chronic pain with increased pain sensitivity [10,11,14], it is difficult to interpret these findings in the context of the handful of other studies that have examined PTSD without pain [15-17], in order to better understand the direct link between pain sensitivity and PTSD.

In addition to the issue of examining pain sensitivity in PTSD with and without chronic pain, the handful of studies on PTSD and pain sensitivity were limited by the use of primarily veteran and male samples with only one type of trauma (i.e., combat) and having participants drawn from a clinical population [15,16,18]. Therefore, the goals of this preliminary study were to address some of the previous limitations by examining the following: 1) examine cold pressor pain ratings at baseline, threshold and tolerance (i.e., perceptual indicators) for community male and female participants with PTSD but without chronic pain compared with participants with other anxiety disorders, and non-anxious controls; and 2) examine time from cold pain threshold to tolerance (i.e., behavioral indicator) for the three groups of participants.

## Methods

### Participants and procedures

Participants with PTSD, with other anxiety disorders, and healthy controls were recruited through flyers and advertisements in the community, physician contacts, and mental health clinics at the Veteran’s Affairs San Diego Healthcare System (VASDHS) and the University of California, San Diego. Potential participants were screened on the telephone to assess initial eligibility (i.e., PTSD, other anxiety disorders, healthy control). Exclusion criteria for all groups were as follows: 1) serious or unstable medical illnesses (e.g., cancer, kidney failure, stroke, cardiovascular disease, seizure, etc.); 2) physical impairments (e.g., blindness, deafness); 3) schizophrenia or other psychotic disorders; 4) current alcohol abuse or dependence as assessed by the Alcohol Use Disorders Identification Test (AUDIT-C) [19]; 5) current drug abuse or dependence through detailed questions; 6) a history of negative reactions to cold temperatures or any conditions such as Raynaud’s disease, temperature-induced urticaria, or other conditions that are exacerbated or influenced by cold temperatures; and 7) a history of chronic pain lasting for more than 6 months based on the Wall and Mezlack’s (1999) [20] definition which considers chronic pain to last 6 months, be ongoing, and not due to life threatening causes. All participants who met the above inclusion and exclusion criteria were invited to participate. For the analyses presented here, we took an additional conservative step by excluding all individuals reporting any pain lasting 1–5 months from the reported analyses rendering our final sample free of any pain other than sporadic pain of less than 1 month duration (e.g., occasional headache, menstrual cramps, etc.). Participants who were determined to be initially eligible based on the telephone screen attended an on-site study visit, where they took part in a cold pressor task and completed a battery of questionnaires. Participants subsequently underwent a structured psychiatric interview. Participants agreed not to take any pain medications for 12 hours prior to the on-site study visit.

Of the 267 individuals who were contacted for a telephone screen, 145 were initially eligible and 120 came in for the lab study and underwent the structured psychiatric interview. Since participants were primarily recruited from the community, their initial enrollment into the study was based on their self-report of symptoms and diagnoses. Once the structured psychiatric interview was completed, there were 59 individuals who did not clearly fit into the diagnostic categories based on our strict inclusion criteria for group placement (e.g., self-reported a diagnosis of PTSD but were not positive for PTSD based on the interview, etc.). Thus, 61 of the 120 met the criteria for PTSD, another anxiety disorder, or were controls, and all of the other inclusion and exclusion criteria to be included in these analyses. The study was approved by the Institutional Review Board of the University of California, San Diego, and the VASDHS Research and Development Committee. Written informed consent was obtained from all participants. Participants received $15 for their time in completing the structured interview and $30 for their time at the study visit. All authors had access to the data.

### Measures

#### Demographics

Demographic information including age, gender, marital status, education, income, and race/ethnicity were obtained as part of the initial telephone screening.

#### Structured psychiatric interview

Participants were administered the PTSD and anxiety disorders modules of the Composite International Diagnostic Interview (CIDI), a structured and lay administered telephone psychiatric interview that assesses for current and historic diagnoses as established in the ICD-10 and DSM-IV [21]. The interview was administered by bachelor level assessors who were trained to administer the interview reliably with other assessors and were supervised by a licensed clinical psychologist, who reviewed the results of each interview. The CIDI is a widely used diagnostic interview in population-based samples and has acceptable reliability and validity in community studies [22,23], and was used to confirm diagnosis and group placement. Individuals who had a primary diagnosis of PTSD based on the DSM-IV criteria were placed in the PTSD group. Individuals who did not meet the criteria for PTSD but met the DSM-IV criteria for another anxiety disorder (i.e., generalized anxiety disorder, social anxiety, obsessive compulsive disorder, specific phobia, and agoraphobia without panic disorder) were placed in the other anxiety group. Individuals who did not meet the criteria for PTSD or any other anxiety disorder were placed in the control group.

#### Self-report questionnaires

The severity of PTSD was assessed with the Posttraumatic Stress Disorder Checklist (PCL). The PCL is a widely used instrument with 17 items designed to assess the extent of overall PTSD symptoms by calculating the sum of all 17 items. Higher scores indicate greater overall PTSD symptom severity. The instrument is highly reliable, with good convergent validity [24]. Internal consistency in our sample was high (Cronbach’s alpha =0.99).

The severity of anxiety symptoms related to generalized anxiety disorder (GAD) was assessed with the GAD-7, a 7-item self-report measure designed for use in primary care settings where the mean of all 7 items was calculated [25]. Higher scores indicate greater severity of GAD symptoms. The instrument is a highly reliable measure with strong convergent validity [26]. High internal consistency was established in our sample (Cronbach’s alpha =0.98).

Symptoms of depression were assessed using the Patient Health Questionnaire (PHQ), a widely used 9-item self-report instrument that evaluates the presence of depression symptoms in the past 2 weeks [27]. Higher sum scores indicate greater depression symptoms. This scale is highly reliable and has good convergent validity [28], with high internal consistency in our sample (Cronbach’s alpha =0.96).

General health status was determined by the widely used Short-Form Health Survey (SF-36) [29]. This is a 36-item self-report measure consisting of eight subscales: physical functioning, physical role limitations, emotional role limitations, mental health, general health, bodily pain, social functioning, and vitality. Raw scores were standardized with higher scores indicating better functioning. The SF-36 is a highly reliable measure with acceptable convergent validity [29]. High internal consistency was established in our sample (Cronbach’s alpha ranging from 0.86 to 0.94).

#### Cold pressor task

Cold pain ratings and time to threshold and tolerance were assessed by the well-validated and widely used cold pressor test in which one side of a container was filled with water that was kept at approximately 1–2°C through the use of ice cubes and a submerged pump in the other side of the container [30]. Participants were asked to place their non-dominant hand and forearm in the water compartment and to indicate when the sensation changed from cold to pain (*threshold*) and when the participant could no longer stand the pain (*tolerance*), upon which they could withdraw their arm from the water. Instructions prior to the task informed participants that they would not keep their arm in the water for longer than 5 min. Participants were instructed to rate their current level of pain intensity and unpleasantness immediately prior to immersing their hand in the water (baseline), at threshold, and at tolerance. The pain intensity and unpleasantness ratings were determined with the Gracely Box Scale which is composed of two separate 21-box numerical descriptor scales: the sensory scale, which assesses pain intensity, and the affective scale, which assesses unpleasantness. Ratings range from 0 (no pain/neutral pleasantness) to 20 (extremely intense pain/very intolerable). The Gracely Box Scales for intensity and unpleasantness were displayed on two separate large posters in the exam room for participants to use as a reference for their ratings. The Gracely Box Scale has been used in both clinical and healthy populations [31,32] and has high reliability and construct validity [33]. The cold pressor protocol was administered by female research staff blind to participant group status.

We examined the linear trajectory of pain ratings from baseline to threshold and tolerance as the perceptual indicator of pain sensitivity. This approach accounts for baseline levels of current pain and unpleasantness ratings prior to the start of the cold pressor task in which a steeper linear trajectory across the cold pressor task would suggest higher cold pain sensitivity (i.e., high evoked pain and unpleasantness ratings at threshold and tolerance relative to baseline rating), while a blunted linear trajectory would suggest lower cold pain sensitivity (i.e., lower evoked pain and unpleasantness ratings at threshold and tolerance relative to baseline rating). Time in seconds from submersion to threshold and to tolerance was measured using a stopwatch. Because the feeling of pain does not begin immediately upon submergence, the difference in time from threshold to tolerance was used as the behavioral indicator of pain sensitivity [34,35].

### Statistical analyses

Data were analyzed using Statistical Package for Social Sciences (SPSS) 16.0 software (IBM, Inc.). An initial set of analyses compared demographic and clinical characteristics according to group status, using one-way analysis of variance (ANOVA) with post hoc contrasts for continuous measures and the chi-square test for the independence of measures for categorical variables. Given our small sample size and the exploratory nature of this study, we determined that correction for multiple comparisons would more likely create a large inflation of type II error and would be an overly conservative approach. One-way ANOVAs were used to compare differences on the cold pressor variables according to gender, veteran status, place of recruitment (clinic versus community), and birth control and pain medication usage. We also examined the Pearson product- moment correlation coefficients among pain and unpleasantness rating variables and the time variables to decide if any of the time variables should be used as covariates in the main study analyses. We then conducted a mixed-design ANOVA with group (three levels: control, anxiety, PTSD) as the between-subjects factor, time point (three levels: baseline, threshold, and tolerance) as the within-subjects factor, and pain and unpleasantness ratings as the response variables, with post hoc contrasts to evaluate group differences. Finally, we used Cox proportional-hazard regression to determine if time from threshold to tolerance differed according to group status. Cox proportional-hazard regression is the appropriate analytic strategy because the time from threshold to tolerance was not normally distributed since individuals kept their arms in the cold pressor for 5 min and therefore never reached their “true” level of tolerance. Resulting hazard ratios examined the rate for reaching pain tolerance from threshold in the PTSD group compared with the other anxiety and control groups. The level of statistical significance was set at *p* <0.05.

## Results

### Participant characteristics

Of the 61 participants who met all of the study criteria, 16 had a primary DSM-IV diagnosis of PTSD; 12 met the DSM-IV criteria for any anxiety disorder other than PTSD (i.e., 42% generalized anxiety disorder, 25% social anxiety, 33% obsessive compulsive disorder, 25% specific phobia, 8% agoraphobia without panic disorder), and 33 did not have PTSD or another anxiety disorder and were included as controls. Of individuals diagnosed with PTSD, 69% also met the criteria for another anxiety disorder (i.e., 38% generalized anxiety disorder, 31% obsessive compulsive disorder, 38% social anxiety, 26% specific phobia). Although these additional diagnoses could likely be ruled out due to a primary diagnosis of PTSD (American Psychiatric Association, 2000), we are unable to do so given that our diagnoses were based on a structured interview and not determined clinically. Only 4 participants (6.3%) were veterans, 11 (18%) were from a mental health clinic setting, and the remaining participants were recruited through flyers and advertisements. In terms of medication usage, 3 participants (4.9%) were taking antidepressant medications, 3 (4.9%) were using other psychotropic medications, 5 (8.2%) were taking pain medication, 2 (3.3%) were taking birth control pills, and 14 (23%) were taking medication for other conditions such as hypothyroidism and allergies.

For the 16 individuals included in the PTSD group, the following trauma types were reported as the most distressing by the participants: 1 (6.3%) had combat-related trauma, 1 (6.3%) witnessed someone being badly injured or killed, 1 (6.3%) experienced rape, 3 (18.8%) experienced sexual molestation, 5 (31.3%) experienced serious physical attack or assault, 3 (18.8%) experienced torture or terrorism, and 2 (12.5%) were threatened by a weapon or held captive. Of these trauma types, the one instance of having witnessed someone being badly injured or killed, all instances of sexual molestation, and one of the instances of serious physical attack or assault were all reported to have occurred before the age of 18 years.

Table 1 presents the demographic characteristics of the entire sample and by group status. Participants were on average 41 years old (SD =13 years); approximately 49% were male, and 62% were White. The PTSD and other anxiety disorder groups had significantly lower rates of a college degree than the control group. There were no other group differences on demographic characteristics.

**Table 1:** **Demographic characteristics of all participants and by group status**

	**All**	**PTSD**	**Anxiety**	**Control**
	***N*** **= 61**	***N*** **= 16**	***N*** **= 12**	***N*** **= 33**
Age, mean (SD)	41 (13)	43 (12)	43 (14)	39 (14)
Male, %	49	47	42	49
Marital status, %				
Single, never married	54	50	58	55
Married/living with partner	20	6	25	24
Divorced/separated/widowed	26	44	17	21
Race, %				
American Indian or Alaskan Native	0	0	0	0
Asian	5	0	0	9
Black or African American	18	25	33	9
Native Hawaiian or Pacific Islander	3	0	0	6
White	62	63	58	64
Other/none reported	12	12	9	12
Hispanic/Latino, %	16	31	17	9
Education, %*				
11th grade or less/not reported	5	13	0	3
12th grade/high school grad or equivalent	13	19	33	3
Some college/technical or vocation grad	38	56	51	24
Bachelor’s degree or higher	44	12	16	70

*p < 0.05.

### Clinical characteristics

Table 2 presents findings on self-reported clinical characteristics by group status. There were greater PTSD symptoms, anxiety symptoms, and symptoms of depression in both the PTSD and other anxiety groups compared to the control group (*p* <0.05 for all measures). As expected, symptoms of PTSD were also significantly higher in the PTSD group compared to the anxiety group (*p* <0.05). Individuals in the PTSD and other anxiety disorders groups exhibited poorer health functioning across all subscales of the SF-36 compared to the control group (*p* <0.05). In addition, the PTSD group exhibited significantly poorer role physical health functioning compared to the other anxiety disorders group (*p* <0.05).

**Table 2:** **Clinical characteristics by group status**

	**PTSD**	**Anxiety**	**Control**	***p*** **value (overall** ***F*** **test)**
	***N*** **= 16**	***N*** **= 12**	***N*** **= 33**	
	**Mean (SD)**	**Mean (SD)**	**Mean (SD)**	
PTSD Checklist	54 (18.5)*^†^	42.1 (19.5)*^∆^	20.8 (8.0)^†∆^	<0.00
Generalized Anxiety Disorder Scale	1.8 (0.8)^†^	1.8 (1.1)^∆^	0.1 (0.3)^†∆^	<0.00
Patient Health Questionnaire	15.1 (7.3)^†^	13.8 (9.4)^∆^	1.2 (2.0)^†∆^	<0.00
Short Form Health Survey				
Physical functioning	79.4 (22.6)^†^	80.8 (24.9)^∆^	95.5 (11.9)^†∆^	0.01
Role physical	57.8 (46.3)*^†^	81.3 (30.4)*	94.7 (15.0)^†^	0.001
Bodily pain	64.3 (24.5)^†^	68.5 (21.1)^∆^	87.8 (14.2)^†∆^	<0.00
General health	60.4 (22.7)^†^	58.8 (19.6)^∆^	82.7 (14.0)^†∆^	<0.00
Vitality	42.2 (21.1)^†^	40.8 (23.8)^∆^	73.3 (11.1)^†∆^	<0.00
Social functioning	53.9 (29.8)^†^	51.0 (33.1)^∆^	97.7 (7.9)^†∆^	<0.00
Role emotional	37.5 (43.7)^†^	38.9 (44.6)^∆^	94.9 (18.9)^†∆^	<0.00
Mental health	50.0 (18.1)^†^	41.0 (23.4)^∆^	86.2 (9.4)^†∆^	<0.00

*p < 0.05 between PTSD and anxiety participants; ^†^ *p* < 0.05 between PTSD and control participants; ^∆^ *p* < 0.05 between anxiety and control participants; PTSD: posttraumatic stress disorder; SD: standard deviation.

### Cold pressor outcomes

There were no significant differences on any of the cold pressor outcomes according to gender (*p* =0.444–0.968), veteran status (*p* =0.458–0.812), place of recruitment (*p* =0.159–0.985), and birth control (*p* =0.393–0.869) or pain medication usage (*p* = .061–0.808). Therefore, data from all participants were analyzed together and the above factors were not used as covariates. However, 2 participants were excluded from the analyses due to faulty equipment at the time of evoked pain testing.

**Figure 1:**
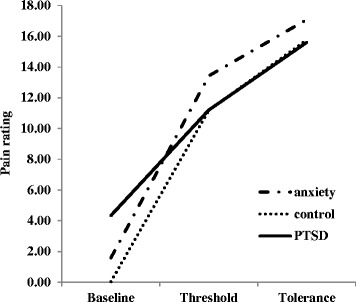
**The linear trajectory of pain ratings in response to cold pressor task across time point by group status.**

Table 3 presents the raw pain and unpleasantness ratings at baseline, threshold, and tolerance and the average times (in seconds) to threshold and tolerance as well as the time from threshold to tolerance by group status. The correlation coefficients for the association between the rating and time variables were not significant; therefore, the time variables were not used as covariates in the mixed-designed ANOVA to examine the trajectory of pain ratings by group status. The mixed-design ANOVA with group (three levels: control, anxiety, PTSD) as the between-subjects factor, time point (three levels: baseline, threshold, and tolerance) as the within-subjects factor, and pain ratings as a response variable found a significant omnibus group by time point interaction (*Wilks’ Lambda F* (4, 110) =3.078, *p* =0.019, ${\eta}_p^2\kern0.5em =\kern0.5em 0.101$). To follow up on this significant omnibus interaction, two contrasts were performed and found to be significant. The linear trajectory across time points differed between PTSD and control groups (*F* (1, 45) =11.20, *p* =0.002, ${\eta}_p^2\kern0.5em =\kern0.5em 0.199$) and also between PTSD and anxiety groups (*F* (1, 25) =4.82, *p* =0.038, ${\eta}_p^2\kern0.5em =\kern0.5em 0.162$). Figure 1 presents the linear trend of reported pain ratings across time points for each group. We then examined unpleasantness ratings and the omnibus group by time interaction was not significant (*Wilks’ Lambda F* (4, 110) =1.885, *p* =0.118, ${\eta}_p^2\kern0.5em =\kern0.5em 0.064$).

**Table 3:** **Pain and unpleasantness ratings and time to threshold and tolerance by group status**

	**PTSD**	**Anxiety**	**Control**
	***N*** **= 15** ^**a**^	***N*** **= 12**	***N*** **= 32** ^**a**^
	**Mean (SD)**	**Mean (SD)**	**Mean (SD)**
Pain ratings			
Baseline	4.33 (4.63)	1.58 (3.29)	.03 (0.18)
Threshold	11.2 (4.13)	13.4 (3.23)	11.2 (4.0)
Tolerance	15.6 (3.29)	17.1 (2.11)	15.7 (0.56)
Unpleasantness ratings			
Baseline	3.2 (3.74)	4.17 (4.13)	.44 (1.21)
Threshold	10.0 (4.05)	12.2 (3.53)	10.4 (3.23)
Tolerance	15.0 (3.54)	15.7 (2.50)	15.2 (3.50)
Time in seconds			
Time to threshold	19.3 (16.8)	26.3 (53.0)	28.6 (47.5)
Time to tolerance	97.0 (116.3)	50.5 (81.3)	66.8 (78.4)
Time from threshold to tolerance	77.7 (104.8)	24.3 (28.6)	38.1 (48.1)

^a^Data from 2 participants (one in the PTSD group and one in control group) are not included due to faulty equipment at the time of testing.

Cox regressions that examined the hazard ratio trajectories for the behavioral pain sensitivity indicator of time to tolerance from threshold by group status were not significant. However, there was a trend (*p* =0.1) that PTSD participants took 2 times longer in reaching tolerance from threshold compared to individuals in the other anxiety group (95% confidence interval =0.84 to 4.6) and 1.3 times longer than individuals in the control group (95% confidence interval =0.6 to 2.6). Although not statistically significant, individuals in the other anxiety group were 1.5 times faster in reaching tolerance from threshold compared to those in the control group (95% confidence interval =0.8 to 3.1).

## Discussion

This preliminary study is one of a handful to examine pain sensitivity in individuals with PTSD, other anxiety disorders, and healthy controls. Using a cold pressor task, we found that individuals with PTSD exhibited a significantly flatter linear trajectory of cold pain ratings over time compared to individuals with other anxiety disorders and control individuals. In addition, individuals with PTSD had a longer cold pain response time to tolerance compared to those with other anxiety disorders, although this difference only approached significance likely due to reduced power. With the exception of PTSD symptoms, individuals with PTSD and other anxiety disorders did not differ on any of the measures of clinical characteristics, and general health functioning, but were significantly worse than individuals in the control group. Finally, there were no differences in any of the cold pressor outcomes based on gender, veteran status, place of recruitment, and birth control or pain medication status. Therefore, our findings suggest that these clinical and demographic characteristics likely may not account for the pattern of findings between PTSD and other anxiety disorders in our sample. Taken together, the flatter trajectory of pain ratings and greater duration from threshold to tolerance of PTSD participants compared to the other groups suggest possibly *lower* cold pain sensitivity for these individuals using both perceptual and behavioral indicators of pain sensitivity.

Similar to previous findings [6,9,36], individuals with PTSD and other anxiety disorders had more severe clinical symptoms across a range of domains including psychiatric symptoms and physical health functioning. Although not a clinical measure of pain, higher baseline pain ratings were reported in individuals with PTSD compared to the other groups, even after specifically excluding individuals who would meet the criteria for a chronic pain diagnosis. These findings are somewhat consistent with previous studies documenting the co-occurrence of PTSD and chronic pain [4,5]. Also consistent with previous research, we found that individuals with PTSD took longer to respond to painful stimuli compared to individuals with other anxiety disorders [18]. However, our findings on perceptual indicators of pain sensitivity are different from the one study which found that individuals with PTSD and chronic pain reported more intense evoked pain ratings compared to those with other anxiety disorders and healthy controls [18], but are consistent with others which found that individuals with PTSD alone reported lower pain ratings compared to healthy individuals after accounting for baseline level of pain [15,16,37,38]. The discrepant findings could be due to previous study’s inclusion of individuals with chronic pain who may be more perceptually sensitive to experimental pain stimuli. Our findings add to the literature by suggesting individuals with PTSD who do not report any chronic pain may have lower pain sensitivity to evoked stimuli even if they report greater baseline pain compared to individuals with other anxiety disorders.

Our findings are also interesting in light of the existing literature documenting increased pain sensitivity in those with chronic pain conditions [10,11], suggesting that there may be different psychological, biological, and physiological mechanisms underlying the processing of painful stimuli in PTSD and chronic pain. In order to examine what is likely a complex set of associations, our study was a preliminary step to determine the level of pain sensitivity in individuals with PTSD only. It would not have been possible to conclude if the findings on evoked pain were due to PTSD or due to chronic pain without excluding participants who also had chronic pain. These findings can inform future research to examine the additive influence of chronic pain on pain sensitivity in individuals with PTSD, and the explanations for why the expression of pain sensitivity may potentially differ across PTSD alone and PTSD with chronic pain. These findings may also potentially shed light on a subtype of PTSD which is free of chronic pain.

Defrin et al. (2008) [18] suggest two plausible mechanisms to explain evoked pain sensitivity alterations in PTSD. In terms of sensory processing of painful stimuli, altered brain processing has been found in individuals with PTSD [15,17,37] suggesting that lowered pain sensitivity in individuals with PTSD could be related to altered brain processes that are also related to avoidance. Individuals with PTSD and those with various pain conditions also have been reported to exhibit basal diminished hypothalamic-pituitary-adrenal (HPA) axis response [39] as well as diminished response to a cold pressor test compared to individuals in a control group [40]. Together, these findings suggest that altered brain processes and dysregulation in HPA axis activity may play a role in the differing sensory processing of pain stimuli in individuals with PTSD compared to other groups.

In terms of the role of emotional experiences in pain processing, several factors related to the emotional experience of PTSD may also influence differential processing of painful stimuli. Individuals with PTSD not only have higher rates of chronic pain [1-3,5,8,37,41] but also exhibit higher anxiety sensitivity [41] and fear of pain [42], which may impact the processing of their traumatic experiences as well as the experience of pain. One potential explanation is that those with PTSD may experience dissociation, involving disruption in body awareness and a detachment from the emotional content of a traumatic experience [43] that may also numb these individuals to physical pain [44]. Future studies should examine what role dissociative and numbing symptoms play in the reduced pain sensitivity seen in individuals with PTSD. Given that similar psychological factors such as fear of pain and pain catastrophizing are related to an increase in pain intensity [45] and emotional distress [46], future research also can examine the role of these and other psychological constructs in the relationship between PTSD and evoked pain sensitivity. Also, there is documented comorbidity of PTSD and depression [47,48] and a mediational role of depression in the relationship between PTSD and pain symptoms [49] that suggest depression as another factor to consider in studies of evoked pain sensitivity with PTSD and chronic pain. Further clarification of this relationship may ultimately shed light on the role of pain sensitivity in the development and maintenance of co-occurring PTSD and chronic pain conditions.

One of the caveats of the present study (and other studies) is that it is impossible to determine the direction of the relationship between evoked pain sensitivity and PTSD or other anxiety disorders. Although lowered pain sensitivity may be an outcome, it might also be a risk factor for the development of PTSD. Given the accumulating findings on lowered evoked pain sensitivity in PTSD, there is a need for future research to examine the role of pain sensitivity itself in the development of co-occurring PTSD and chronic pain, as the relationships may be complex. One model that addresses the potential underlying relationships between these problems is the shared vulnerability model, which proposes that shared individual difference factors, such as anxiety sensitivity and sympathetic dysregulation, influence the development of these disorders [3]. The mutual maintenance model asserts that physiological, affective, and behavioral components exacerbate and maintain symptoms of PTSD and pain [42].The perpetual avoidance model further asserts dysfunctional cognitive processing following trauma can lead to an increase in psychological and physiological arousal and therefore greater behavioral avoidance that maintains both PTSD and pain [50]. Further, there is a dearth of research examining the co-occurrence of pain and other anxiety disorders and a need to better understand this co-occurring relationship beyond PTSD and pain [51]. Future research to examine the temporal order of lowered evoked pain sensitivity in the development of these conditions can shed some light on their co-occurrence.

PTSD is unique in that it requires the occurrence of a serious adverse life event prior to symptom development. PTSD also is characterized by numbing, alienation, and detachment which are generally associated with depression symptoms [52]. However, symptoms of PTSD include hypervigilance and reactivity which are similar to other anxiety disorders. The symptom characteristic of PTSD and a growing literature on the distinction between PTSD and other anxiety disorders [52-55] has led to a substantial change in the DSM-V, by removing PTSD from the list of anxiety disorders and placing it within a new group of disorders entitled “Trauma and Stressor Related Disorders” [52]. Our findings on lowered evoked pain sensitivity in PTSD compared to other anxiety disorders may also provide potential support for a likely distinction between trauma-related conditions and anxiety disorders and also suggest that the mechanisms underlying the processing of painful stimuli in PTSD are likely different from the mechanisms involved in pain processing in anxiety. Future studies should further examine whether our findings are unique to PTSD or are better accounted for by the experience of a traumatic event.

This study has several limitations. First, we had a relatively small sample size which may have hampered our ability to detect significant differences in the behavioral indicator of pain sensitivity, although we were able to detect significant differences in the perceptual indicators. Further, we did not conduct a medical record review to confirm the diagnosis of PTSD, or other anxiety disorders nor did we examine inter-rater reliability of our PTSD and anxiety disorder groups. Therefore, our findings should be replicated with a larger sample of participants with physician-confirmed diagnoses distinguishing PTSD from other anxiety disorders and other comorbidities and examine the inter-rater reliability of these diagnoses. It is important for future research to examine whether PTSD exclusively accounts for these differences in pain sensitivity or whether presence of multiple diagnoses leads to lowered pain sensitivity. Second, while we did not find any gender, veteran status, place of recruitment, and birth control or pain medication usage differences on the evoked pain measures, given our sample size, future studies should examine the potential influence of these factors on evoked pain stimuli in larger samples. A recent review of sex differences in pain experiences suggests increased pain sensitivity and risk for clinical pain in women compared to men [56]. Therefore, additional large-scale studies are needed to examine sex differences in evoked pain sensitivity in PTSD that will consider factors such as the impact of the women’s hormonal cycle, genetic factors, and pain coping on women’s responses to evoked pain stimuli as potential explanatory mechanisms of sex differences. In addition, our sample size precluded us from examining the potential role of physical impairment on pain sensitivity in individuals with PTSD and other anxiety disorders. Third, our assessment of pain sensitivity focused on cold pain and may not generalize to pain sensitivity in other domains such as heat or pressure nor to pain sensitivity paradigms like the tender points exam that are able to localize specific or regional areas of pain. However, our findings are consistent with previous studies that have examined heat pain sensitivity [15,16,37]. A fourth issue is that our anxiety disorders group was heterogeneous; future studies should examine whether the relationship between other anxiety disorders and evoked pain sensitivity differs by type of anxiety disorder. Fifth, the study procedures were administered by female research staff, which may have influenced the responses of male participants on the study assessments. Additionally, participants were told that they could remove their hand from the cold pressor task after a time limit, possibly creating an expectancy effect. Therefore, future studies using the cold pressor task methodology should address these concerns to minimize any bias in participant responses or ceiling effects. Finally, individuals were included in this study by their self-report that they had not been diagnosed or treated for chronic pain. However, it is possible that participants may have been experiencing other conditions in which pain is a feature that were not reported at the time of the experiment. Therefore, it is recommended that future studies use physician-confirmed diagnoses or medical records to confirm any chronic or acute pain and other diagnoses. We consider the present study a first step in examining the relationship between PTSD only and experimental pain sensitivity. Future larger studies should compare pain sensitivity in those with PTSD, chronic pain, and PTSD comorbid with chronic pain.

## Conclusions

In conclusion, our findings suggest that although individuals with PTSD but without chronic pain report significantly more clinical symptoms and issues with health functioning than controls, when examining both perceptual and behavioral indicators of pain sensitivity, they appear to exhibit lower perceived cold pain sensitivity compared to individuals in the control group and those with other anxiety disorders. These findings suggest a need to further examine mechanisms that may explain the differing relationship of PTSD and other anxiety disorders with pain sensitivity and how these findings relate to existing models of co-occurring PTSD and chronic pain. A greater understanding of these relationships can better explicate the development, maintenance, and ultimately treatment of co-occurring PTSD, anxiety, and chronic pain.

## Abbreviations

ANOVA: analysis of variance
AUDIT-C: alcohol use disorders identification test
CIDI: composite international diagnostic interview
GAD: generalized anxiety disorder
PHQ: patient health questionnaire
PTSD: posttraumatic stress disorder
PCL: posttraumatic stress disorder checklist
SF-36: short-form health survey
SPSS: statistical package for social sciences
SD: standard deviation
VASDHS: Veteran’s Affairs San Diego Healthcare System
